# Supplementary material for: Behavior of Male Gamete Fusogen GCS1/HAP2 and the Regulation in Arabidopsis Double Fertilization
Source: Biomolecules. 2023 Jan 20;13(2):208. doi: 10.3390/biom13020208 (PMC9953686; doi:10.3390/biom13020208)
Supplement: Supplementary file 1 [file biomolecules-13-00208-s001.zip › Supplementary files-2131145.pdf]

Supplementary Figure S1

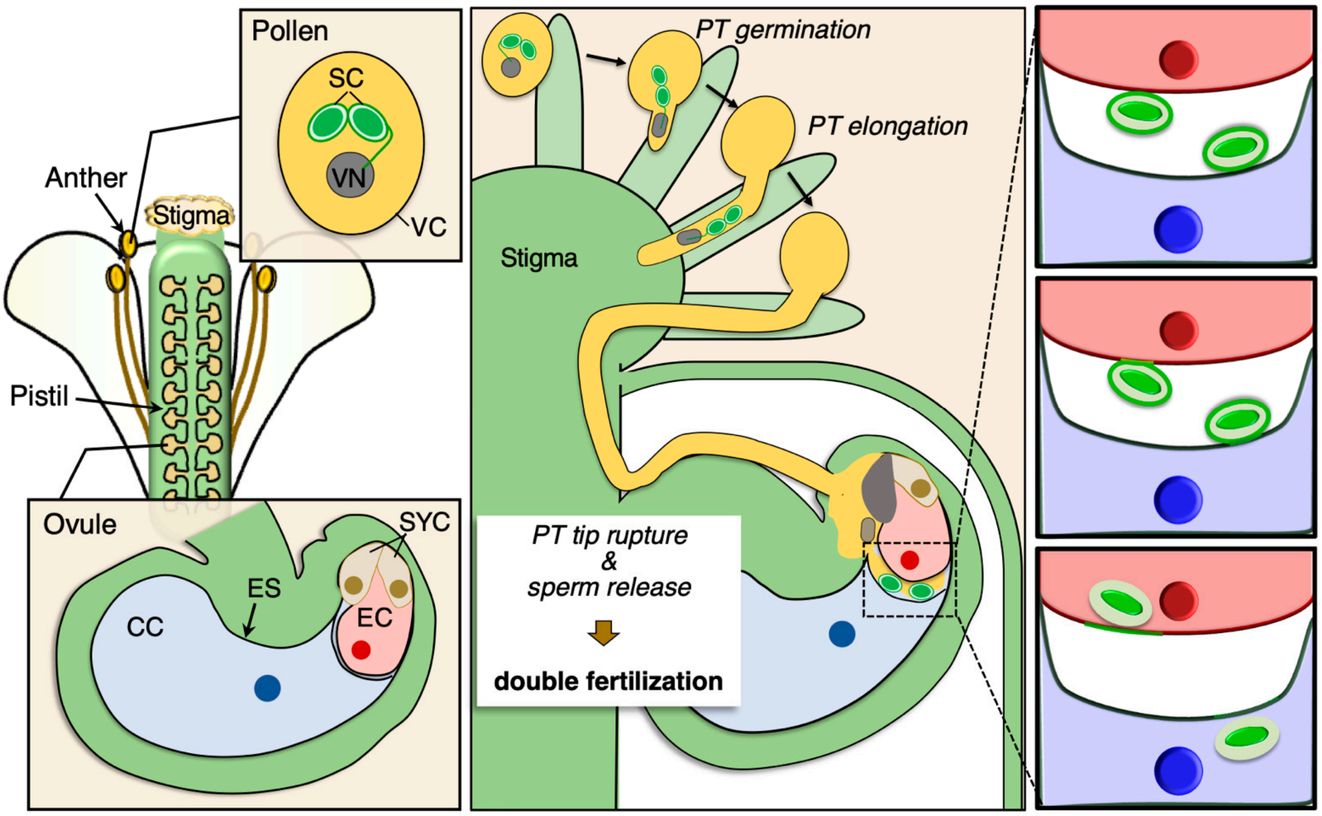

Supplementary Figure S1. Illustration of sexual reproduction processes observed in *Arabidopsis,* a model flowering plant.

The left drawing represents the anatomy of an *Arabidopsis* flower, comprised of four sepals, four petals, six stamens and a pistil. The sepals, a petal, and two stamens are omitted here. Pollen grain is produced in the anther. Around 50 ovules are arranged into two rows within a pistil. At the mature stage, each ovule contains an embryo sac (ES), which is comprised of an egg cell (EC), a central cell (CC), and two synergid cells (SYC). Pollen is comprised of three cells; a pair of sperm cells (SC) is enclosed with a vegetative cell (VC). A cytoplasmic tail protrudes from one of the SCs, tethering to the VC nucleus (VN). The middle panel indicates the SCs delivery by a pollen tube. Upon pollination, pollen germinates a pollen tube (PT). The PT penetrates the pistil tissue and elongates the tip delivering a pair of sperm cells toward an ES. The PT is guided toward the ES because of the attraction by SYCs; then, one of the SYCs accepts the PT. Afterward, the tip of PT ruptures and the sperm cells are released into an ES. The SYC that accepted a PT degenerates (colored in gray). The released two sperm cells are located at the boundary of EC and CC. The right panels show the flow of double fertilization processes (top to bottom). After the SCs attach to the EC and CC, plasma membrane fusion occurs [38], followed by plasmogamy. The process from sperm cell release to plasmogamy progresses in several minutes [46]. If double fertilization is not achieved, the 2nd PT is attracted by the remaining SYC to recover the fertilization failure [3]. All ovules in a pistil accept at least one PT by around 10 hours after pollination.

Supplementary Figure S2

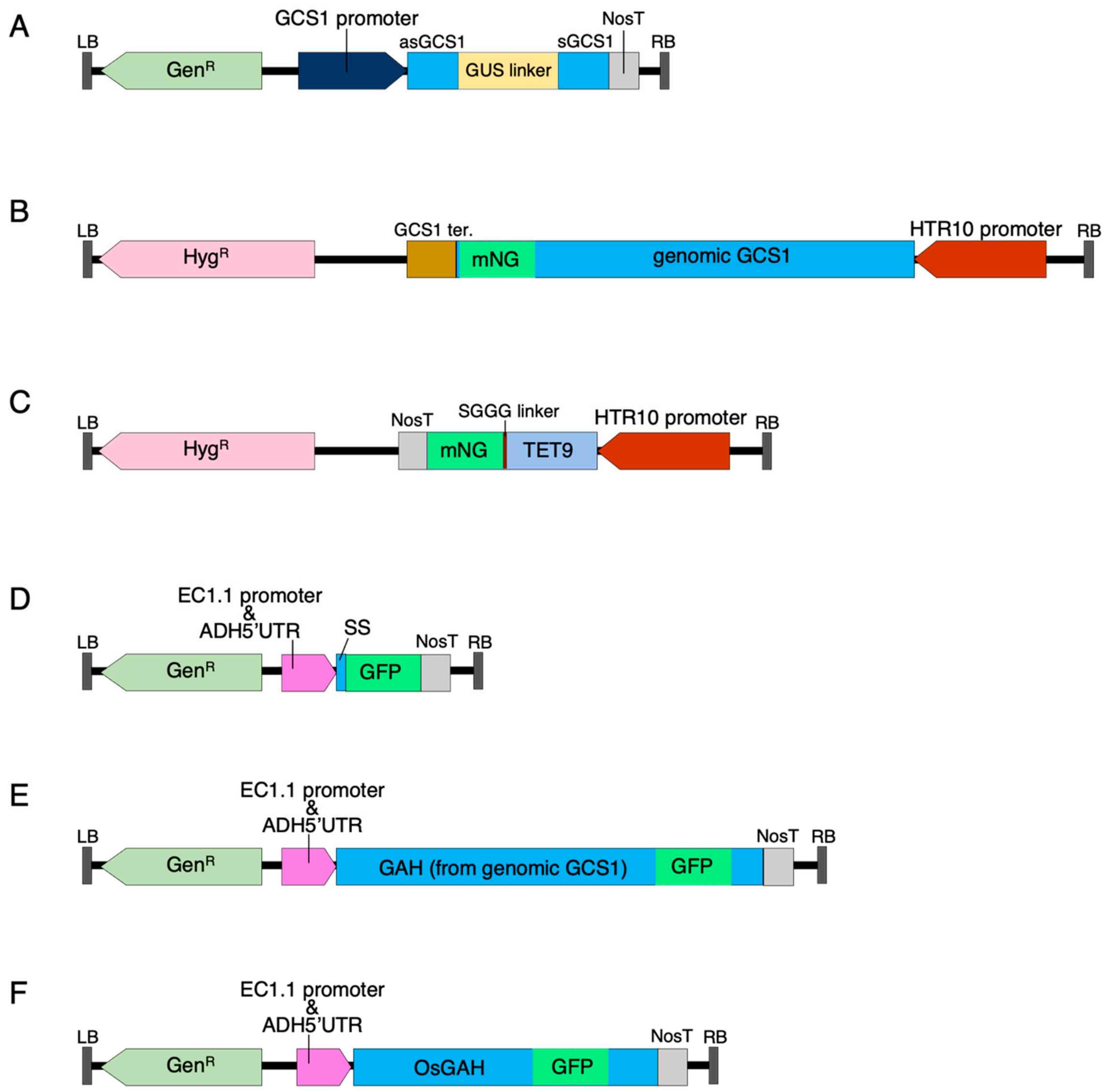

Supplementary Figure S2. Schematic diagrams of T-DNA region constructed in this study.
(A) GCS1_RNAi construct. as: antisense; s: sense. **(B, C)** GCS1-mNG (B) and TET9-mNG (C) constructs. ter.: terminator. **(D-F)** ssGFP (D), GAH (E), and OsGAH (F) constructs. SS: signal peptide sequence from GCS1/HAP2. Gentamycin-resistant (GenR) and hygromycin-resistant (HygR) genes are under the control of CaMV35S promoter and the poly(A) signal sequences. NosT: NOS terminator; LB; left border; RB: right border.

Supplementary Figure S3

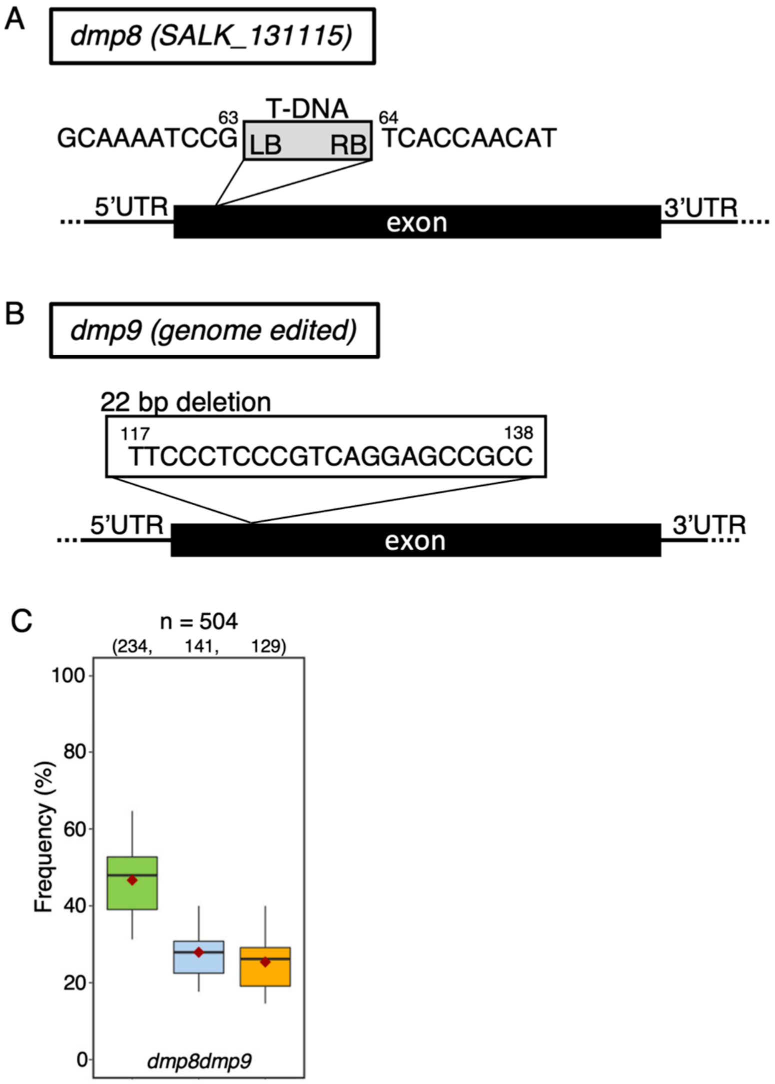

Supplementary Figure S3. Genomic information and phenotypes of the *dmp8dmp9* plant produced in this study.
(A) The *dmp8* (SALK_13115) includes T-DNA insertion between the 63rd and 64th nucleotides in the exon. **(B)** In the *dmp9* plant, 22 bp deletion from 117th to 138th was generated by CRISPR/Cas9 genome editing system in this study. By crossing each homozygous mutant line, *dmp8dmp9* with background of HTR10mRFP was produced. **(C)** Frequencies of each seed development phenotype observed in the produced *dmp8dmp9* plants. Green, pale-blue, and orange boxplots reflect normal, undeveloped, and aborted seed phenotypes, respectively. Seeds (n = 504) from 10 siliques were counted, and the numbers in parenthesis indicated at the top of each boxplot are the total counts of each phenotype. The horizontal bar and a violet diamond on each boxplot represent the median and the mean, respectively.

Supplementary Figure S4

A

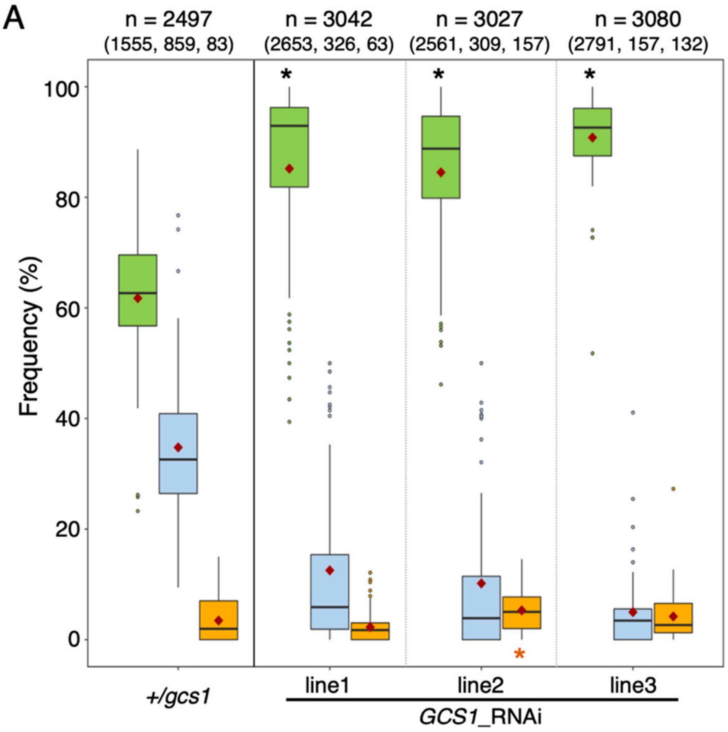

B

*GCS1*_RNAi	HTR10mRFP Normal	*+/gcs1* Aborted
line 1	1.68×10^{-08}*	2.98×10^{-01}
line 2	1.25×10^{-10}*	3.84×10^{-03}*
line 3	2.45×10^{-09}*	1.90×10^{-01}

Supplementary Figure S4. Seed development observed in the *GCS1*_RNAi homozygous T3 plants.
(A) Frequencies of each seed development phenotype observed in +/*gcs1*, and *GCS1*_RNAi plants. Green, pale-blue, and orange boxplots reflect normal, undeveloped, and aborted seed phenotypes, respectively. Seeds from 10 siliques were counted per plant, and 6 plants for every transgenic line were analyzed independently. The numbers indicated at the top of each transgenic line are the total counts of the seeds (at 1st line) and each phenotype (at 2nd line in parenthesis). The horizontal bar and a violet diamond on each boxplot represent the median and the mean, respectively. The dots associated with each boxplot represent the outliers in the counted data. The black asterisks above the green boxplot represent the significant differences in normal seed phenotype relative to the HTR10mRFP (Figure 1A data). The orange asterisk below orange boxplot in *GCS1*_RNAi line 2 represent a significant difference in the aborted phenotype relative to the +/*gcs1* plant. Significant differences were analyzed with the Wilcoxon rank-sum test ($p < 0.05$).
(B) *P*-values detected in the comparisons of each *GCS1*_RNAi transgenic line with HTR10mRFP and +/*gcs1* plants in normal and aborted seed development phenotypes, respectively. *$p < 0.05$.

Supplementary Figure S5

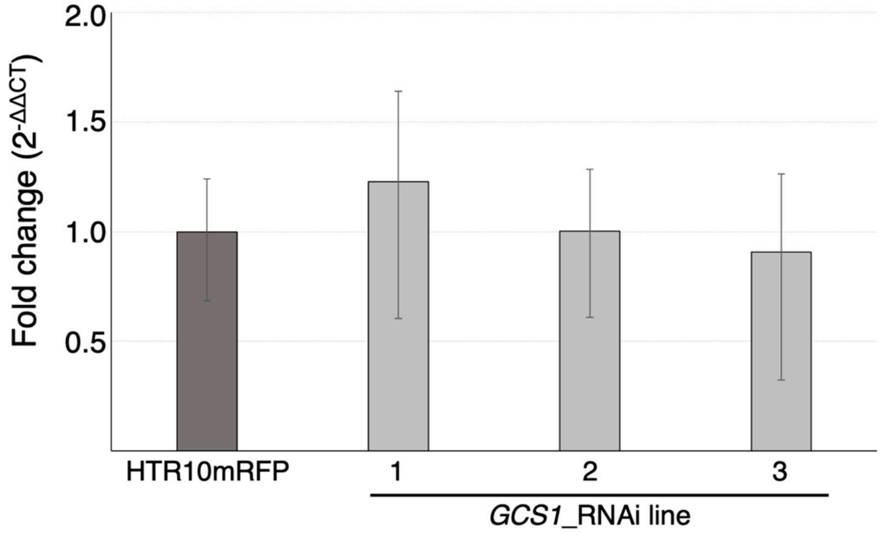

Supplementary Figure S5. Quantitative real-time PCR analysis of *GCS1* in pollen population from *GCS1*_RNAi T3 homozygous plants (lines 1-3).

The pollen from the original HTIR10mRFP plant was used as the control. The relative quantification is indicated as the fold change calculated with the average ΔΔCT value (n = 3). Bars on each bar graph represent the standard errors.

Supplementary Figure S6

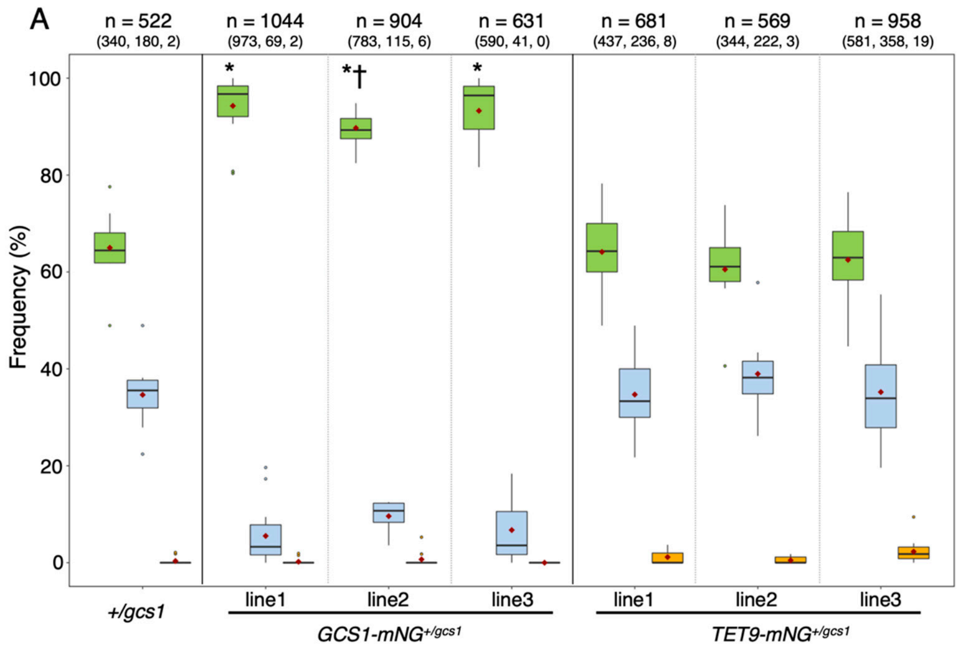

B

Phenotype	Genotype	Compared Genotype (1)	Compared Genotype (2)
		+/gcs1	HTR10mRFP
Normal	*+/gcs1*		9.41×10^{-07}
	GCS1-mNG line 1	1.19×10^{-05}**	2.65×10^{-02}*
	GCS1-mNG line 2	3.85×10^{-05}**	2.41×10^{-07}**
	GCS1-mNG line 3	3.85×10^{-05}**	2.15×10^{-02}*
	TET9-mNG line 1	8.39×10^{-01}	1.98×10^{-07}**
	TET9-mNG line 2	1.30×10^{-01}	2.19×10^{-06}**
	TET9-mNG line 3	5.00×10^{-01}	4.80×10^{-08}**
Undeveloped	*+/gcs1*		1.97×10^{-07}**
	GCS1-mNG line 1	1.20×10^{-05}**	1.89×10^{-04}**
	GCS1-mNG line 2	5.50×10^{-05}**	9.14×10^{-08}**
	GCS1-mNG line 3	8.08×10^{-05}**	4.91×10^{-04}**
	TET9-mNG line 1	8.39×10^{-01}	4.45×10^{-08}**
	TET9-mNG line 2	1.52×10^{-01}	4.44×10^{-07}**
	TET9-mNG line 3	9.19×10^{-01}	1.18×10^{-08}**
Aborted	*+/gcs1*		8.15×10^{-01}
	GCS1-mNG line 1	6.15×10^{-01}	4.07×10^{-01}
	GCS1-mNG line 2	8.06×10^{-01}	6.40×10^{-01}
	GCS1-mNG line 3	1.67×10^{-01}	1.20×10^{-01}
	TET9-mNG line 1	1.47×10^{-01}	8.21×10^{-02}
	TET9-mNG line 2	7.41×10^{-01}	8.14×10^{-01}
	TET9-mNG line 3	9.73×10^{-03}**	1.06×10^{-03}**

Supplementary Figure S6. Functional complementation of the *gcs1* mutant phenotype with the GCS1-mNG. **(A)** Frequencies of each seed development phenotype observed in +/*gcs1*, GCS1-mNG and TET9-mNG plant lines. Green, pale-blue, and orange boxplots reflect normal, undeveloped, and aborted seed phenotypes, respectively. The numbers indicated at the top of each transgenic line are the total counts of the seeds (at 1st line) and each phenotype (at 2nd line in parenthesis). The horizontal bar and a violet diamond on each boxplot represent the median and the mean, respectively. The dots associated with each boxplot represent the outliers in the counted data. The asterisk and the dagger indicate the significant differences relative to the +/*gcs1* and HTR10mRFP plant (Figure 1) ($p < 0.01$). **(B)** *P*-values detected in the comparisons of each transgenic lines in each seed development phenotype. Statistical analyses were performed using the Wilcoxon rank-sum test. *$p < 0.05$, **$p < 0.01$.

Supplementary Figure S7

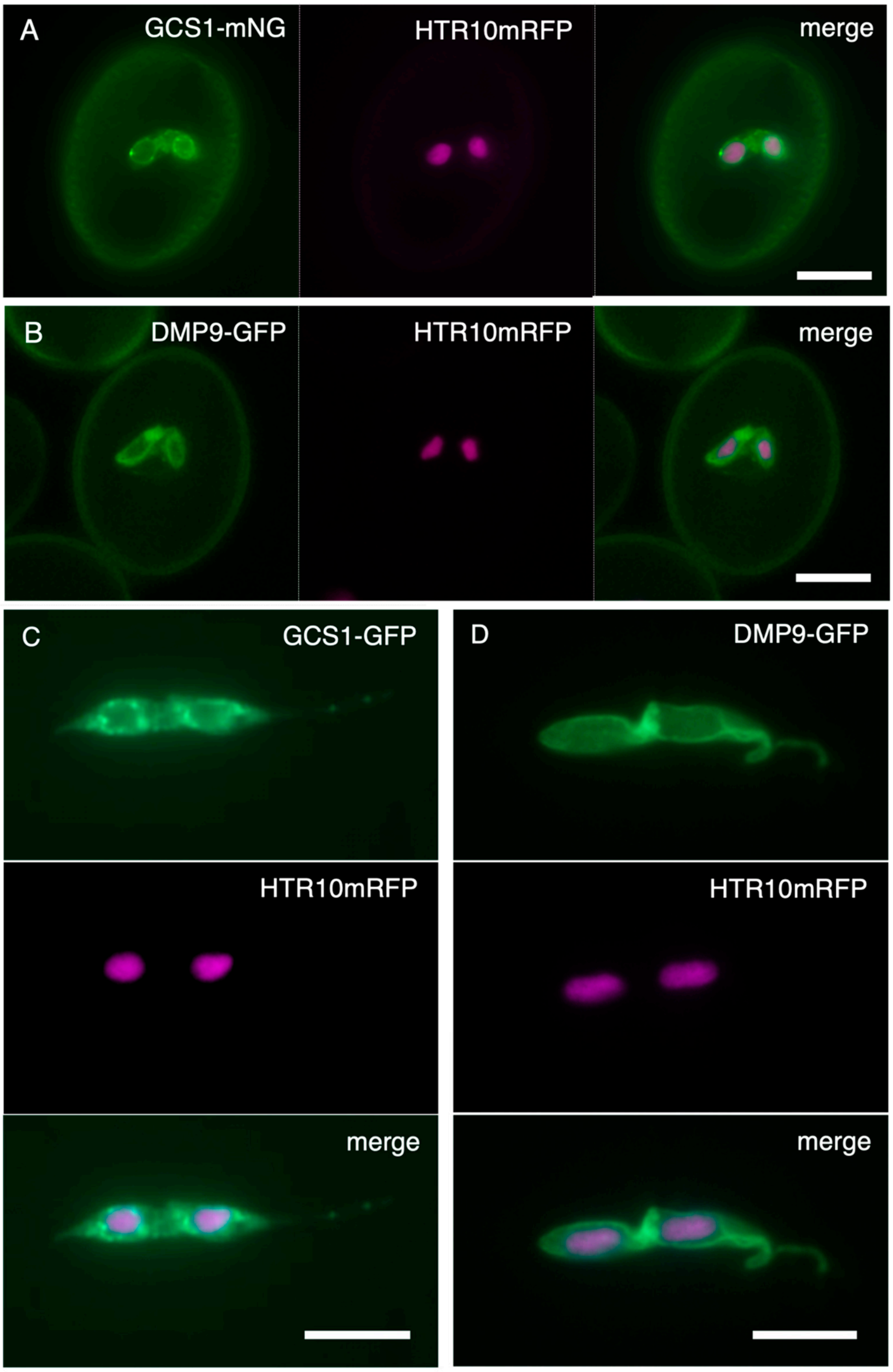

Supplementary Figure S7. GCS1 distribution patterns in pollen and a pollen tube.
(A) GCS1-mNG in which two sperm cells are at the same focus plane (left panel). **(B)** DMP9-GFP, as the example of plasma membrane protein expressed in the sperm cells. (A, B) middle panel: the sperm nuclei labeled with HTR10mRFP; right panel; merged image of mNG and HTR10mRFP. **(C, D)** Fluorescence images of GCS1-GFP (GPP, a functional GCS1 variant) [31] (C) and DMP9-GFP (D) in a pollen tube (top panels). The middle and the bottom panels represent the sperm nuclei labeled with HTR10mRFP and merged images, respectively. Bars = 10 μm.

Supplementary Figure S8

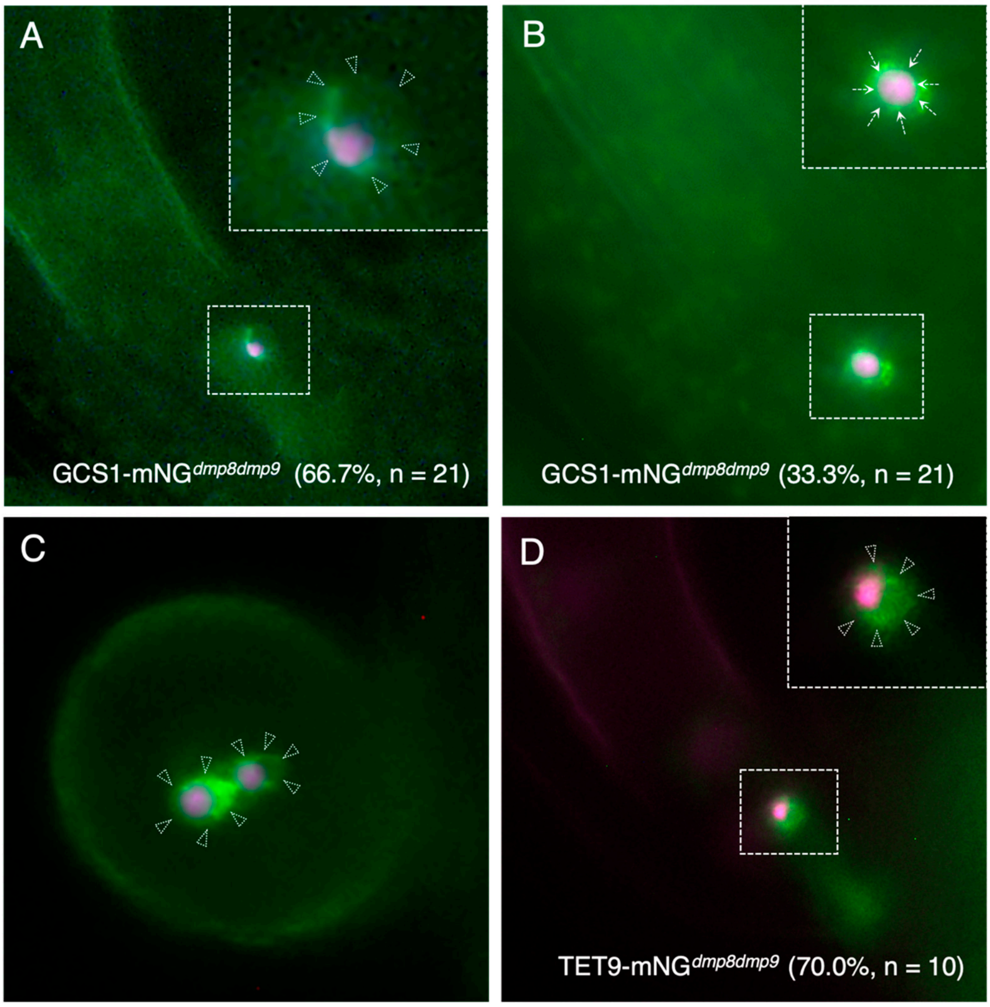

Supplementary Figure S8. GCS1/HAP2 and TET9 distribution patterns in the sperm cells.
The sperm nuclei were visualized with RFP (magenta). **(A, B)** GCS1-mNG signals in an arrested *dmp8dmp9* sperm. (A) A pattern of linear signal (dashed triangles) with a spatial gap from the sperm nucleus. (B) Another pattern of GCS1-mNG showing the perinuclear signal (dashed arrows). The upper-right box in each photo is the magnification of the dashed area. **(C)** TET9-mNG distribution in the sperm cells in pollen. The signal detected with a spatial gap from sperm nuclei was observed (dashed triangles), reflecting the plasma membrane. **(D)** TET9-mNG signal in the arrested *dmp8dmp9* sperm in an embryo sac. Signals with a spatial gap from the nucleus were detected. The upper-right box is the magnification of the dashed area.

Supplementary Figure S9

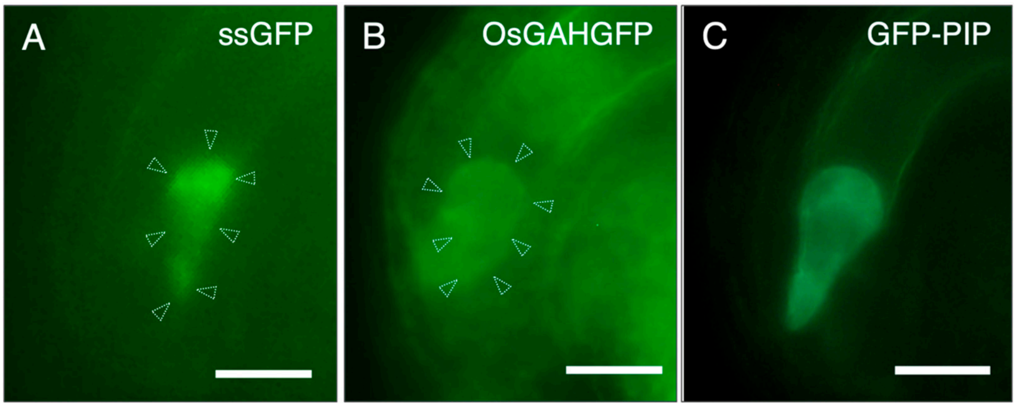

Supplementary Figure S9. Expressions of GCS1/HAP2 variants in the egg cell.
(A, B) The GFP signals in egg cell (positions marked with dashed triangles) from the ssGFP (A) and OsGAH (B) expressed by *EC1*promoter and *AtADH*5'-UTR translational enhancer, respectively. **(C)** The egg cell expressing *GFP-PIP2a* driven by *EC1*promoter and *AtADH*5'-UTR, as a membrane marker. As shown by the higher backgrounds, the GFP signals from the egg cell expressing ssGFP, GAH (Figure 3C), and OsGAH were relatively weak and showed vague outlines compared to that of GFP-PIP. These characteristics may reflect the secretion of these proteins from the egg cell due to the lack of transmembrane.

Supplementary Figure S10

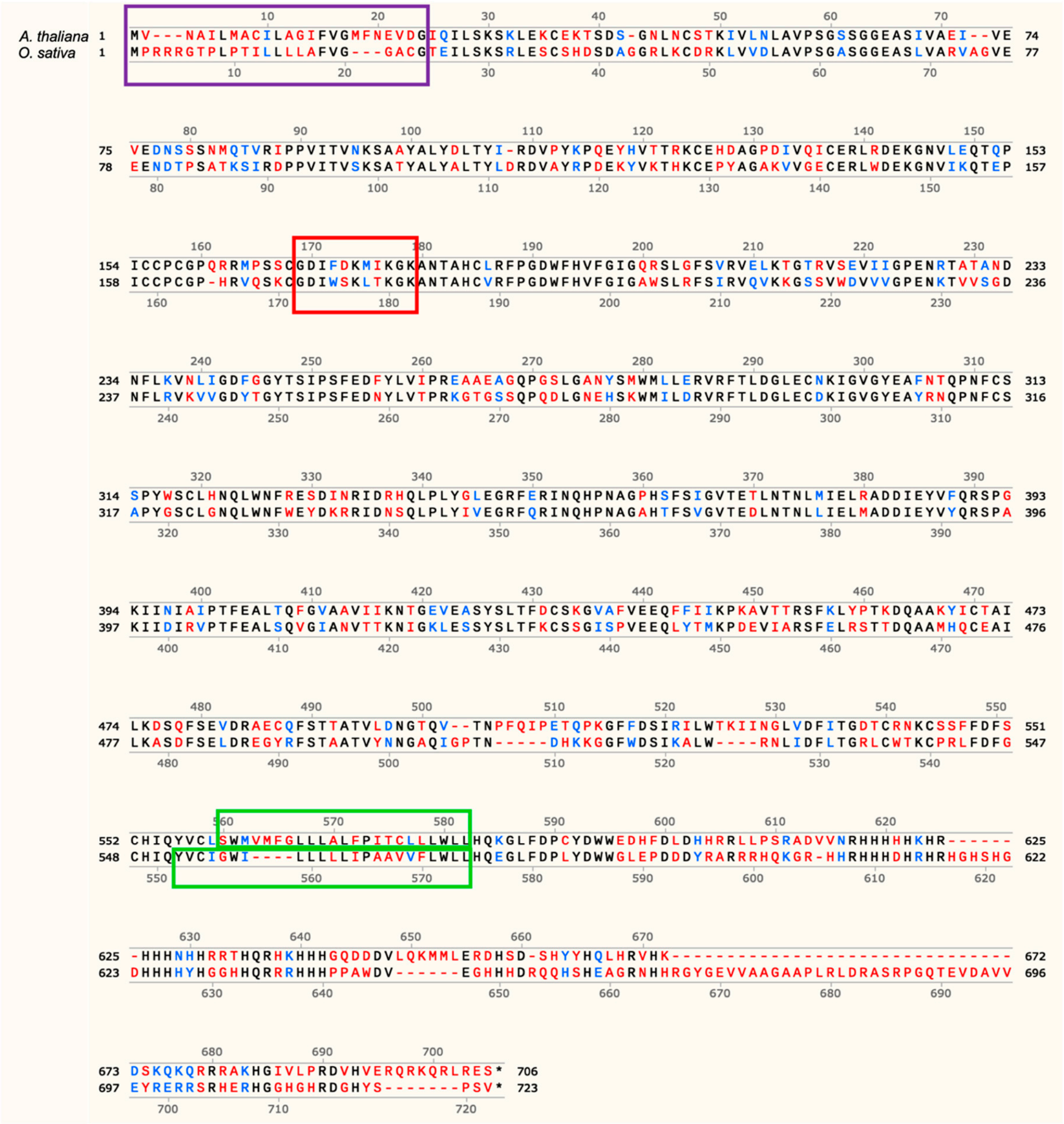

Supplementary Figure S10. Amino acid sequence comparison between the *Oryza sativa* and *Arabidopsis thaliana* GCS1/HAP2.
Residues in blue and red represent similar and different characteristics, respectively. Sequences enclosed with the purple and green boxes are the signal sequences and the transmembrane regions, respectively. Amino acid residues in the red box correspond to the αF region critical for membrane insertion and interchangeable between Arabidopsis and rice [22].

Supplementary Table S1. Primers used in this study. Nucleotides underlined represent the restriction enzyme recognition site.

Primer name	Sequence (5′ to 3′)	Application
KpnI-5'UTR-GCS1	CGGTACCAGCAGAGCACATCTTATCAATTTC	*GCS1*_RNAi
5'UTR-GCS1-BamHI	CGGATCCTCTTCTTGAAGAACTGCTTTCTTC	*GCS1*_RNAi
GCS1-BamHI	CGGATCCTTTGCGCCTAAGCTCCCTGG	*GCS1*_RNAi
XbaI-GCS1	GTCTAGAGTGAACACGATGCTGGACCG	*GCS1*_RNAi
XbaI-GUSlinker	GTCTAGAATCTACCCGCTTCGCGTCGG	*GCS1*_RNAi
GUSlinker-SalI	CGTCGACCGAGTGAAGATCCCTTTCTT	*GCS1*_RNAi
SalI-GCS1	CGTCGACGTGAACACGATGCTGGACCG	*GCS1*_RNAi
GCS1-PstI	GCTGCAGTTTGCGCCTAAGCTCCCTGG	*GCS1*_RNAi
PstI-NosTer	GCTGCAGGATCGTTCAAACATTTGGCA	*GCS1*_RNAi
NosTer-HindⅢ	CAAGCTTGATCTAGTAACATAGATGAC	*GCS1*_RNAi
GCS1qRTf	CGCCGGAATTTTCGTCGGAATG	qRT-PCR for *GCS1*
GCS1qRTr	ACTGGGAACAGCGAGATTCAAGAC	qRT-PCR for GCS1
AteIFG4f	CGGCGATGTTCTTGGGAGTG	qRT-PCR for GCS1
AteIFG4r	CCGGTTAGGTGCATGAGGTTTG	qRT-PCR for GCS1
dmp9_GE(HTR10)_F	GGATTTCCTTTTACCCAAATCCACCGG	*dmp9* selection
dmp9_GE(HTR10)_R	GGCGGCTCCTGACGGGAGGG	*dmp9* selection
Tail_LB1st	AACACTCAACCCTATCTCGGGC	*dmp8* selection
DAU2-ORFfor	ATGGAGAAAACAGAGGAAAGCGTC	DMP8&9 CDS
dmp8_rev	TTATGTAGACATGCATCCGACACC	DMP8 CDS
dmp9_rev	ACCAGTCATGCAACCAACACCATA	DMP9 CDS
Nco_inf_EC1.1_F	CTTATGATTTCTTCGGTTTCAAGAT	GAH
Nco_inf_EC1.1_R	TGTGATTGTGATGTATTCTCAACAGATTGATAAGGT	GAH
Nco_inf_AtADH_F	TACATCACAATCACACAAAACTAACAAAAG	GAH
Nco_inf_AtADH_R	ATCGCGTTCACCATGGTATCAACAGTGAAGAACTTGCTTT	GAH
inf_GAH_For	ATGGTGAACGCGATTTTAATGGCT	GAH
inf_GAH_Rev_Pst	ATGTTTGAACGATCCTGCATTAACTCTCACGTAGTCTTTGTTTCC	GAH
Nco_inf_35S_F	CTGCTGCGTACCATGGTCGATCGACAGATCTGC	GAH
Nco_inf_35S_R	CGAAGAAATCATAAGATTGCGTTGCGCTCACTG	GAH
SS_Eco_inf_GFP for	GAAGTCGACGGAATTATGGTGAGCAAGGGCGAGGA	ssGFP
GFPrev_forSSinf	TTACTTGTACAGCTCGTCCATGCCG	ssGFP
GFP3'_inf_NosTfor	GAGCTGTACAAGTAAGATCGTTCAAACATTTGGCAATAAAGTTTC	ssGFP
inf_NosTrev_MauBI	AGATGACACCGCGCGCGATAATTT	ssGFP
inf_pPZP_Sac_EC1p	ATGATTACGAATTCGAGCTCCTTATGATTTCTTCGGTTTCAAGAT	OsGAH
inf_adh_R_Xba_OsGCS	CGACGACGAGGCATTCTAGATATCAACAGTGAAGAACTTGCTTT	OsGAH

Supplementary Table S2. *P*-values detected in the comparisons of HTR10mRFP, +/*gcs1*, and *GCS1*_RNAi plant lines in each seed development phenotype.

Phenotype	Compared genotypes		P-value
	Genotype (1)	Genotype (2)	
Normal	HTR10mRFP	+/*gcs1*	2.39×10^{-11}**
	GCS1_RNAi line 1	HTR10mRFP	3.90×10^{-08}**
	GCS1_RNAi line 2	HTR10mRFP	2.92×10^{-11}**
	GCS1_RNAi line 3	HTR10mRFP	1.75×10^{-09}**
	GCS1_RNAi line 1	+/*gcs1*	1.38×10^{-07}**
	GCS1_RNAi line 2	+/*gcs1*	3.76×10^{-03}**
	GCS1_RNAi line 3	+/*gcs1*	4.36×10^{-09}**
Undeveloped	HTR10mRFP	+/*gcs1*	1.10×10^{-11}**
	GCS1_RNAi line 1	HTR10mRFP	2.74×10^{-08}**
	GCS1_RNAi line 2	HTR10mRFP	1.05×10^{-08}**
	GCS1_RNAi line 3	HTR10mRFP	3.07×10^{-07}**
	GCS1_RNAi line 1	+/*gcs1*	9.96×10^{-08}**
	GCS1_RNAi line 2	+/*gcs1*	2.53×10^{-04}**
	GCS1_RNAi line 3	+/*gcs1*	1.40×10^{-09}**
Aborted	HTR10mRFP	+/*gcs1*	5.76×10^{-01}
	GCS1_RNAi line 1	HTR10mRFP	1.31×10^{-02}*
	GCS1_RNAi line 2	HTR10mRFP	2.98×10^{-09}**
	GCS1_RNAi line 3	HTR10mRFP	4.37×10^{-08}**
	GCS1_RNAi line 1	+/*gcs1*	2.60×10^{-02}*
	GCS1_RNAi line 2	+/*gcs1*	9.79×10^{-10}**
	GCS1_RNAi line 3	+/*gcs1*	2.64×10^{-08}**

The data were analyzed using the Wilcoxon rank-sum test. *$p < 0.05$, **$p < 0.01$.

Supplementary Table S3. Observed sperm nuclear signal patterns in an embryo sac from *ssGFP* and *GAH* plant lines.

Genotype	Line	Sperm nucleus signal morphology		
		Two dispersed	One condensed	Two condensed
ssGFP	1	96.3<br>(78)	0<br>(0)	3.7<br>(3)
	2	94.1<br>(95)	0<br>(0)	5.9<br>(6)
GAH	2	50.6<br>(42)	8.4*<br>(7)	41.0<br>(34)
	3	50.0<br>(22)	11.4*<br>(5)	38.6<br>(17)

The upper and lower (in parentheses) numbers in each cell indicate frequencies (%) and the counted number of ovules, respectively. The ovules dissected from pistils at 7-9 hours after pollination were observed. In this evaluation, the ovules with over two signals were excluded from the count. The Chi-square tests were performed for the "one condensed signal" cases, and the asterisks indicate that the significant differences were detected to that of each ssGFP line ($p < 0.05$).
